# A real-world pharmacovigilance analysis of acute renal failure associated with sacubitril/valsartan based on FDA Adverse Event Reporting System (FAERS)

**DOI:** 10.1371/journal.pone.0334402

**Published:** 2025-10-15

**Authors:** Shiyu Wang, Jinglun Lai, Jiahao Chen, Qingmao Luo, Wenyan Yi, Yingyu Lai

**Affiliations:** 1 Department of Pharmacy, The People’s Hospital of Hezhou, Hezhou, China; 2 Hezhou Food and Drug Evaluation & Inspection Center, Hezhou, China; 3 Office of Good Clinical Practice Institutional, The People’s Hospital of Hezhou, Hezhou, China; University of British Columbia, CANADA

## Abstract

**Background:**

The renal safety profile of sacubitril/valsartan remains debated in clinical practice. To address this uncertainty, we conducted a pharmacovigilance analysis of acute renal adverse events associated with sacubitril/valsartan using the FDA Adverse Event Reporting System (FAERS) database.

**Methods:**

Using data from the FAERS database, we conducted an observational pharmacovigilance study from the first quarter (Q1) of 2015 to the fourth quarter of 2024 (Q4). The disproportionality analysis was performed using four algorithms: the reporting odds ratio (ROR), the proportional reporting ratio (PRR), the Bayesian confidence propagation neural network (BCPNN), and the multi-item gamma Poisson shrinker (MGPS).

**Results:**

A total of 16,144,939 adverse events (AEs) were available on FAERS database, among these, 132,255 cases were associated with sacubitril/valsartan, 2,205 cases related to narrow acute renal failure (SMQs) and 3,232 cases with broad SMQs. And sacubitril/valsartan and acute renal adverse showed significant association both in narrow SMQs (ROR = 2.37; PRR = 2.34, χ2 = 1703.73; EBGM05 = 2.26) and the broad SMQs (ROR = 2.55, PRR = 2.51, χ2 = 2941.80, EBGM05 = 2.43). A total of 10 (19.23% of total) positive signals were obtained using the above four algorithms. Median age was 71 years, time to onset was within 30 days, and hospitalization rates of acute renal failure outcomes were 14.33% (narrow SMQs) and 30.69% (broad SMQs).

**Conclusion:**

The widespread use of sacubitril/valsartan has raised safety concerns, particularly regarding acute renal failure. Our study, leveraging real-world FAERS data, systematically analyzed this association, providing new evidence on acute renal failure risks.

## Introduction

Sacubitril/valsartan, recognized as the inaugural angiotensin receptor-neprilysin inhibitor (ARNI), obtained its initial approval from the Food and Drug Administration (FDA) approval in 2015 as a first-line treatment in patients with heart failure with reduced ejection fraction (HFrEF). Subsequently, in 2017, the European Medicines Agency (EMA) granted the first global regulatory authorization for its application in managing essential hypertension in adults [1]. Contemporary clinical guidelines endorse its use (Class 1) it for HFrEF, citing its efficacy in decreasing both mortality and the risk of hospitalization [2,3]. While sacubitril/valsartan has demonstrated superior effectiveness compared to conventional therapies in lowering rates of heart failure-related hospital admissions, cardiovascular mortality, and overall mortality, these substantial clinical advantages are inevitably accompanied by a profile of drug-related adverse events (AEs).

As it is well established that Angiotensin-Converting Enzyme Inhibitors (ACEIs) and Angiotensin II Receptor Blockers (ARBs) are associated with an increased risk of acute renal failure. However, potential for additional neprilysin inhibition to further elevate the risk of acute renal events remains a subject of ongoing debate in clinical practice [4]. Large-scale randomized clinical trials (RCTs) have demonstrated that sacubitril/valsartan offers superior cardiovascular and renal benefits compared to ACEIs or ARBs in patients with heart failure (HF) and chronic kidney disease (CKD) [5–7], and it has been shown to exert favorable effects on renal outcomes in heart failure populations [8,9]. In contrast, findings from a separate study indicated that sacubitril/valsartan exerted a comparable impact on renal function in heart failure patients with an ejection fraction of 45% or greater, without demonstrating significant renal protection or benefit [10]. Consequently, despite the renal protective effects reported in prior clinical trials, concerns regarding drug-induced renal impairment remain prevalent in clinical practice. It is important to highlight that the acute hemodynamic changes triggered by sacubitril/valsartan may result in early modifications of renal function, emphasizing the need for vigilant monitoring to reduce the risk of drug-induced acute kidney injury (AKI) [11].

The prevailing uncertainty has contributed to physician hesitancy in prescribing ARNIs, despite their well-established cardiovascular benefits [1]. Limited research exists regarding the incidence of acute renal failure associated with sacubitril/valsartan, and the underlying pathophysiological mechanisms remain inadequately understood. This gap in knowledge may adversely affect patient adherence to treatment regimens or result in severe and potentially irreversible clinical outcomes. Given the widespread use of sacubitril/valsartan, post-marketing pharmacovigilance studies are urgently needed to evaluate acute renal failure risks and enhance patient safety. This study aimed to detect pharmacovigilance signals of acute renal adverse events associated with sacubitril/valsartan using the FDA Adverse Event Reporting System (FAERS).

## Materials and methods

### Data sources and procedures

Utilizing data extracted from the FAERS database, we conducted an observational pharmacovigilance study spanning from the first quarter (Q1) of 2015 through the fourth quarter of 2024 (Q4). Adverse event symptoms were categorized employing the Medical Dictionary for Regulatory Activities (MedDRA, Version 27.0), specifically utilizing Standardized MedDRA Queries (SMQs) related to acute renal failure, alongside all preferred terms (PTs) designated as either “narrow” or “broad” in association with adverse events. The time to onset determined by calculating the interval between date of the adverse event date (event_dt) and the medication start date (start_dt). Cases exhibiting input errors or implausible date values were excluded from the analysis. A comprehensive database encompassing all FDA-approved brand names, generic names, and experimental codes for sacubitril/valsartan was utilized.

### Data cleaning

Two-step data cleaning was conducted before the analysis. First, sort by CASEID, FDA_DT, and the original fields in accordance with the FDA’s suggested procedure for eliminating duplicate reports. If two reports have the same CASEID, keep the one with the higher FDA_DT value; if the CASEID and FDA_DT are the same, choose the one with the highest initial value. Additionally, just the most current, duplicate-free version of each instance was taken away. Second, only AE patients with documented “suspect” drug roles were included in the drug event combination of sacubitril/valsartan and acute renal failure; those with “concomitant” or “interacting” roles were eliminated.

### Acute renal failure (SMQs)

This study employed a systematic search strategy utilizing Standardized MedDRA Queries (SMQs) with both narrow and broad preferred terms to identify acute renal failure cases within adverse event reports coded in MedDRA version 27.0.

The narrow PTs used were: “acute kidney injury”, “acute phosphate nephropathy”, “anuria”, “azotaemia”, “continuous haemodiafiltration”, “dialysis”, “foetal renal impairment”, “haemodialysis”, “haemofiltration”, “neonatal anuria”, “nephropathy toxic”, “oliguria”, “peritoneal dialysis”, “prerenal failure”, “renal failure”, “renal failure neonatal”, “renal impairment”, “renal impairment neonatal”, “subacute kidney injury”.

The broad PTs used were: include the narrow PTs and “albuminuria”, “blood creatinine abnormal”, “blood creatinine increased”, “blood urea abnormal”, “blood urea increased”, “blood urea nitrogen/creatinine ratio increased”, “creatinine renal clearance abnormal”, “creatinine renal clearance decreased”, “creatinine urine abnormal”, “creatinine urine decreased”, “crystal nephropathy”, “fractional excretion of sodium”, “glomerular filtration rate abnormal”, “glomerular filtration rate decreased”, “hypercreatininaemia”, “hyponatriuria”, “intradialytic parenteral nutrition”, “kidney injury molecule-1”, “nephritic syndrome”, “nephritis”, “neutrophil gelatinase-associated lipocalin increased”, “oedema due to renal disease”, “protein urine present”, “proteinuria”, “renal function test abnormal”, “renal transplant”, “renal tubular disorder”, “renal tubular dysfunction”, “renal tubular injury”, “renal tubular necrosis”, “tubulointerstitial nephritis”, “urea renal clearance decreased”, “urine output decreased”.

### Statistical analysis

Statistical analysis and data processing were carried out using R-4.4.3. Four methods were used for the disproportionality analysis: the reporting odds ratio (ROR), the proportional reporting ratio (PRR), the Bayesian confidence propagation neural network (BCPNN), and the multi-item gamma Poisson shrinker (MGPS). Continuous variables were expressed as mean ± standard deviation for normally distributed data. Moreover, an event was classified as a potential adverse reaction if it met the positivity criterion in any of the analysis methods. The specific formulas and thresholds used for disproportionality analysis are provided in S1 and S2 Tables.

## Results

### Descriptive analysis

**Fig 1:**
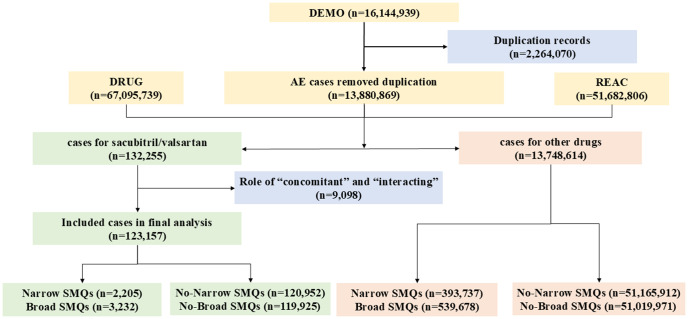
The selection algorithm flowchart.

From Q1 2015 to Q4 2024, a total of 16,144,939 adverse events (AEs) were available on FAERS database and 13,880,869 AEs which were deduplicated cases were extracted. Among these, 132,255 cases were associated with sacubitril/valsartan. After excluding concomitant and interacting drugs, 123,157 cases were included in the final analysis. Additionally, 2,205 cases related to narrow demented AEs and 3,232 cases with broad demented AEs were documented. All these data are shown in Fig 1.

**Fig 2:**
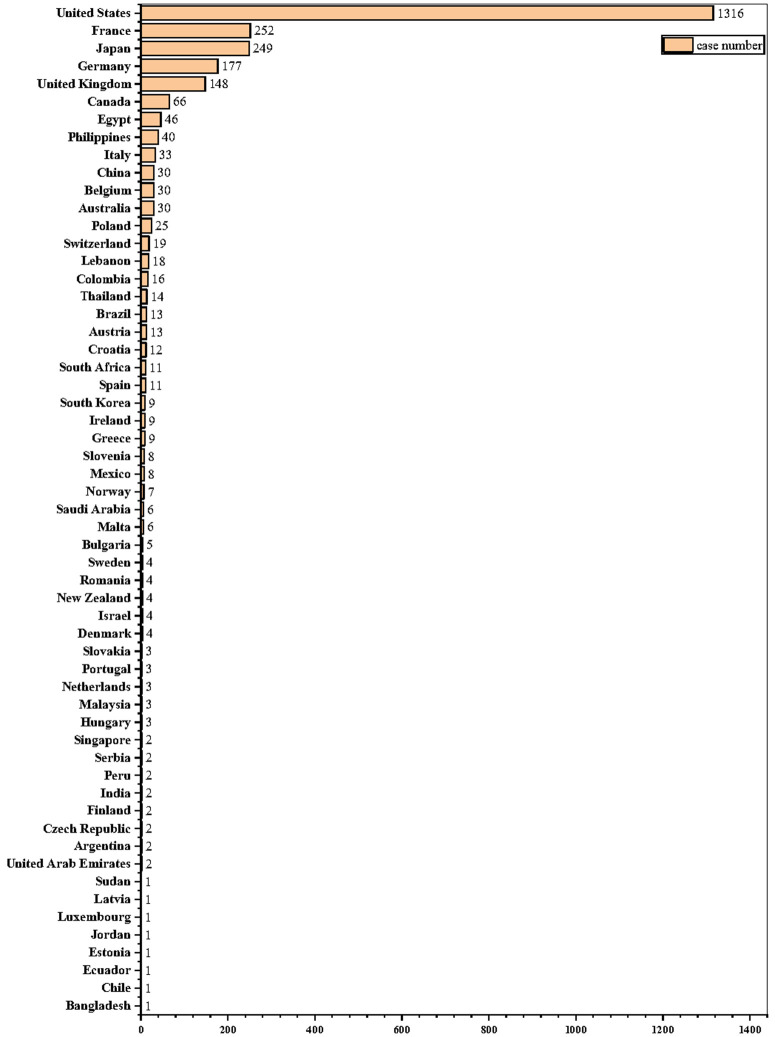
Worldwide distribution of reported cases by country/area.

The demographics of cases of acute renal failure associated with sacubitril/valsartan are described in detail in Table 1. Male reports were more frequent in all cases. Both the narrow and broad definitions were consistent with a median age of 71 years for patients with recorded age data. Documented weight measures were absent from most case reports. Furthermore, acute renal failure associated with sacubitril/valsartan was mainly reported by physicians (35.33%) on narrow SMQs and non-health professionals (42.23%) on broad SMQs, most of the cases are reported after 2018. The US was the main reporter country (48.83%), as detailed in Fig 2.

**Table 1:** Baseline characteristics of acute renal failure associated with sacubitril/valsartan.

Characteristics	Reports *n* (%)	
	Narrow SMQs	Broad SMQs
All reports	2205	3232
**Gender**		
Female	449 (20.36)	672 (20.79)
Male	1132 (51.34)	1678 (51.92)
Unknown or missing	624 (28.30)	882 (27.29)
**Age (year)**		
< 18	1 (0.05)	2 (0.06)
18 ≤ and < 65	257 (11.66)	383 (11.85)
65 ≤ and < 75	317 (14.38)	450 (13.92)
≥ 75	409 (18.55)	590 (18.25)
Unknown or missing	1221 (55.37)	1807 (55.91)
Median	71 (59–83)	71 (58–83)
**Weight (kg)**		
< 50	16 (0.73)	25 (0.77)
50 ≤ and < 100	358 (16.24)	467 (14.45)
≥ 100	95 (4.31)	131 (4.05)
Unknown or missing	1736 (78.73)	2609 (80.72)
Median	84 (60–108)	84 (60–109)
**Reporter**		
**Health‐care professional**		
Physician	779 (35.33)	1201 (37.16)
Pharmacist	125 (5.67)	146 (4.52)
Other	15 (15.15)	1365 (42.23)
Unknown or missing	374 (16.96)	520 (16.09)
**Reported year**		
2015	34 (1.54)	50 (1.55)
2016	199 (9.02)	321 (9.93)
2017	349(15.83)	519 (16.06)
2018	364 (16.51)	536 (16.58)
2019	279 (12.65)	413 (12.78)
2020	190 (8.62)	299 (9.25)
2021	230 (10.43)	323 (9.99)
2022	192 (8.71)	298 (9.22)
2023	244 (11.07)	320 (9.90)
2024	124 (5.62)	153 (4.73)

### Disproportionality analysis

The disproportionality signals identified through quantitative analysis are compiled in Table 2. The ROR (95% Cl) was 2.37 (2.27–2.47), the PRR (χ2) was 2.34 (1703.73), and the EBGM05 was 2.26 for narrow acute renal failure-related AEs associated with sacubitril/valsartan, demonstrating as positive signals. Similarly, the broad SMQs were reported with positive signals, the ROR (95% Cl) was 2.55 (2.46–2.64), the PRR (χ2) was 2.51 (2941.80), and the EBGM05 was 2.43.

**Table 2:** Presents the overall disproportionality analysis results, demonstrating significant signal detection across all algorithms employed.

SMQs	Cases	ROR(95%Cl)	PRR (χ2)	EBGM05
**Narrow SMQs**	2205	2.37 (2.27 - 2.47)	2.34 (1703.73)	2.26
**Broad SMQs**	3232	2.55 (2.46 - 2.64)	2.51 (2941.80)	2.43

**Fig 3:**
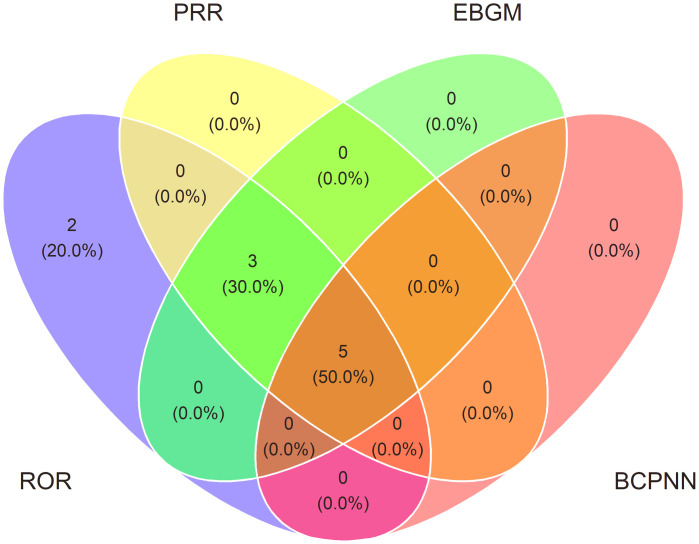
Venn diagram of overlapping results identified by four algorithms.

The ROR/PRR/BCPNN/EBGM method was used to analyze all the PTs for acute renal injury, and a total of 10 (19.23% of total) positive signals were obtained, including “glomerular filtration rate decreased”, “blood creatinine increased”, “blood creatinine abnormal”, “anuria”, “azotaemia”, “acute kidney injury”, “renal impairment”, “renal function test abnormal”, “renal failure”, and “blood urea increased”. These signals are shown in Table 2. Among the ten PTs, five PTs suggested high reliability by four methods, three PTs suggested high reliability by three methods, and two PTs suggested high reliability by one method. These signals are shown for sacubitril/valsartan in Table 3 and Fig 3.

**Table 3:** Results of overall disproportionality analysis.

PTs	N	Scope	ROR	PRR	χ^2^	IC025	ROR(95%Cl)	EBGM (EBGM05)	ROR(lower limit of 95% CI > 1, N ≥ 3)	PRR (PRR ≥ 2, χ2 ≥ 4, N ≥ 3)	MGPS (EBGM05 > 2)	BCPNN (IC025 > 0)
Glomerular filtration rate decreased	111	Broad	4.33	4.32	280.75	0.43	4.33 (3.59-5.22)	4.29 (3.67)	**Y**	**Y**	**Y**	**Y**
Blood creatinine increased	752	Broad	6.39	6.36	3346.88	0.98	6.39 (5.94-6.87)	6.28 (5.91)	**Y**	**Y**	**Y**	**Y**
Blood creatinine abnormal	36	Broad	4.86	4.86	109.06	0.6	4.86 (3.5-6.75)	4.81 (3.66)	**Y**	**Y**	**Y**	**Y**
Anuria	46	Narrow	3.51	3.51	81.96	0.14	3.51 (2.63-4.7)	3.49 (2.74)	**Y**	**Y**	**Y**	**Y**
Azotaemia	21	Narrow	3.62	3.61	39.39	0.18	3.62 (2.35-5.56)	3.59 (2.51)	**Y**	**Y**	**Y**	**Y**
Acute kidney injury	1158	Narrow	2.86	2.84	1374.57	−0.17	2.86 (2.7-3.03)	2.83 (2.69)	**Y**	**Y**	**Y**	N
Renal impairment	512	Narrow	2.98	2.97	667.2	−0.1	2.98 (2.73-3.25)	2.96 (2.75)	**Y**	**Y**	**Y**	N
Renal function test abnormal	31	Broad	3.13	3.13	44.67	−0.03	3.13 (2.2-4.46)	3.12 (2.32)	**Y**	**Y**	**Y**	N
Renal failure	447	Narrow	1.61	1.61	103.06	−0.98	1.61 (1.47-1.77)	1.61 (1.49)	**Y**	N	N	N
Blood urea increased	37	Broad	1.62	1.62	8.79	−0.97	1.62 (1.17-2.24)	1.62 (1.24)	**Y**	N	N	N

The intersections represent the shared ROR/PRR/BCPNN/EBGM among algorithms. 10 signals (19.23% of total) were identified by all four methods (e.g., “Anuria”, “Azotaemia”, “Blood creatinine abnormal”, “Blood creatinine increased”, “Glomerular filtration rate decreased”), suggesting high reliability.

### Time to onset

**Fig 4:**
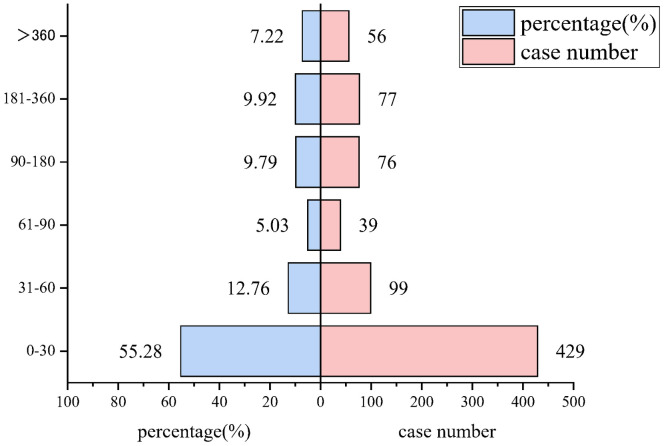
The time to onset of sacubitril/valsartan‐related broad PTs.

Onset time was analyzed using reports in which both drug_start_time and event_time were documented. Most of the AEs (55.28%, 429 cases) in broad SMQs occurred within 30 days after taking sacubitril/valsartan in those with complete onset dates, in Fig 4.

Onset time was analyzed using reports in which both drug_start_time and event_time were documented.

### Outcomes of acute renal failure

As detailed in Table 4, hospitalization rates for sacubitril/valsartan-associated acute renal failure outcomes were 14.33% (narrow SMQs) and 30.69% (broad SMQs). In contrast, life-threatening events (1.86% vs 3.09%), death (1.81% vs 6.99%), and disabling outcomes (0.82% vs 0.87%) demonstrated lower frequencies under both SMQs classifications, respectively.

**Table 4:** The serious outcomes for acute renal failure in sacubitril/valsartan.

Serious outcome	Narrow SMQs, N = 2205, n (%)	Broad SMQs, N = 3232, n (%)
Death	40 (1.81)	226 (6.99)
Life-threatening	41 (1.86)	100 (3.09)
Hospitalization	316 (14.33)	992 (30.69)
Disabling	18 (0.82)	28 (0.87)
Others	239 (10.84)	1504 (46.53)

## Discussion

Sacubitril/valsartan is recommended as first-line therapy for HFrEF patients due to its demonstrated reductions in cardiovascular mortality and hospitalization risks. While existing research has predominantly focused on its applications in heart failure with preserved ejection fraction (HFpEF) [12,13], hypertension [14], and post-myocardial infarction management [15], data regarding its renal effects remain limited. Despite robust evidence supporting its efficacy and expanding indications, real-world adoption has been slower than anticipated. Although clinical trials and observational studies have primarily evaluated safety concerns such as hypotension [16], renal dysfunction [8,17,18], hypokalemia [19], and angioedema [20], the risk of acute renal failure remains controversial. To our knowledge, this represents the first real-world global study utilizing the FDA Adverse Event Reporting System (FAERS) database to investigate sacubitril/valsartan-associated acute renal failure, providing critical post-marketing safety insights. Our key findings include:

First, both the broad (51.92%) and narrow (51.34%) SMQs showed a male preponderance among patients with acute renal failure linked to sacubitril/valsartan. This masculine preponderance most likely results from the interaction of two important factors: (i) Although heart failure is equally common in both sexes overall, there are notable differences in the phenotypic distribution between the sexes. According to Lenselink C [21] study, male sex was an independent predictor of the development of HFrEF in patients with ST-elevation myocardial infarction (STEMI). Women with HFpEF are almost twice as likely to suffer heart failure as those with HFrEF [22]. This difference may be related to the different effects of sex hormones on the cardiovascular system. Consequently, female patients are often excluded for the above reasons. (ii) Low baseline blood pressure may increase the risk of hypotension in women on sacubitril/valsartan sodium [23]. Clinicians frequently express concern about hypotensive risks when initiating sacubitril/valsartan, which may lead to the low utilization rate of it in female patients with heart failure.

Second, both broad and narrow acute renal failure (SMQs) were significantly correlated with sacubitril/valsartan, according to this real-world pharmacovigilance investigation. We compared the acute renal failure signal levels based on PTs following further analysis. Five of the 10 PTs had a stronger signal than the others. These included the broad SMQs of decreased glomerular filtration rate, increased blood creatinine, and abnormal blood creatinine, and the narrow SMQs of anuria, and azotemia. Sacubitril/valsartan’s dual pharmacological action causes vasodilation and increases natriuretic peptide levels by blocking angiotensin II type 1 receptors (AT1) and inhibiting neprilysin (NEP) at the same time. Although this process explains its therapeutic advantages, the sudden change in hemodynamics might cause temporary hypotension, especially in individuals who have already developed an intolerance to Renin-Angiotensin System (RAS) inhibitors (ACEIs/ARBs). Particularly when therapy is started or doses are increased, this sudden drop in systemic blood pressure may jeopardize renal perfusion pressure and cause acute renal failure [11,24,25]. After starting medication, a small percentage of patients had temporary drops in eGFR (>15%), which were probably caused by hemodynamic-mediated drops in intraglomerular pressure [17]. When starting therapy particularly in high-risk groups, such as elderly patients and those with pre-existing chronic kidney disease (CKD), enhanced renal function monitoring is advised.

Third, current evidence indicates sacubitril/valsartan-associated acute kidney injury risk predominantly occurs during initial treatment (days to weeks), particularly in RAS inhibitor-naive patients and those with baseline hypotension or renal impairment [11,24,17]. Comparably, within 30 days of starting sacubitril/valsartan therapy, 55.28% of the patients in our analysis experienced acute renal failure. Additionally, we discovered that the percentage of reported acute renal failure after receiving sacubitril/valsartan rose in 2017–2018 and then fell over the following six years, confirming that acute renal failure was acknowledged as the medication was used more widely. The majority of studies do not precisely define the time at which acute kidney damage begins, even if the precise at-risk period changes depending on patient factors.

Finally, regarding the outcomes of acute renal failure, the present study showed that acute renal failure associated with sacubitril/valsartan contributed to more hospitalizations than death, life-threatening events, and disabling conditions. In addition, we compared the differences in acute renal failure outcomes among specific PTs with significant signals and found that acute kidney injury was the most frequently reported AE. These findings suggest that there is a relatively high frequency of sacubitril/valsartan-related acute renal failure. However, there is a low risk of death, life-threatening outcomes, and disabling outcomes, reflecting that this drug is safe, and with the wider application was the acute renal failure was recognized as controllable. The evidence suggests that it is not prudent to stop sacubitril/valsartan therapy because of unwarranted worries about the medication’s broader use and the known acute renal failure. Cardiovascular doctors should be aware of the possibility of acute renal failure when using valsartan and sacubitril in clinical settings. Consultation with a nephrology department is required in situations of severe acute renal failure.

### Limitations

Although FAERS offers a worldwide surveillance capability, its usefulness is limited by basic pharmacoepidemiologic constraints: risk quantification is impossible in the absence of denominator data, and spontaneous reporting results in incomplete ascertainment. Because of its passive surveillance architecture, the FAERS database records observed temporal connections but is unable to prove a direct causal link between sacubitril/valsartan and acute renal damage. Therefore, to look into causal relationships, well planned clinical investigations are required. Even though percentage imbalance analyses (such ROR and PRR) are frequently employed, confounding variables can affect the results.

## Conclusion

Safety concerns have been raised by the extensive usage of sacubitril/valsartan, particularly in acute renal failure adverse events. Our results offered fresh empirical support for the safety of sacubitril/valsartan in the treatment of acute renal failure. We thoroughly and methodically examined the connection between sacubitril/valsartan and acute renal failure by using the FAERS database. Managing acute renal failure brought on by sacubitril/valsartan requires early detection and prompt action. To completely identify the dangers involved, establish causal links, and elucidate its underlying mechanisms, more research is required.

## Supporting information

S1 Table Two-by-two contingency table for disproportionality analyses. (DOCX)

S2 Table Four major algorithms used for signal detection. (DOCX)
